# It’s Complicated: Rapid Publication of Genomes in *Microbiology Resource Announcements* Can Be Both Part of the Problem and the Solution to Fungal Taxonomic Resolution

**DOI:** 10.1128/MRA.00458-21

**Published:** 2021-12-02

**Authors:** Bradley Stevenson, Blake Stamps

**Affiliations:** a Department of Microbiology and Plant Biology, University of Oklahoma, Norman, Oklahoma, USA; b 711th Human Performance Wing, Airman Systems Directorate, Air Force Research Laboratory, Wright-Patterson AFB, Ohio, USA; c UES Inc., Integrative Health and Performance Sciences Division, Dayton, Ohio, USA; Vanderbilt University

## REPLY

In a recent letter to the editor, Houbraken et al. (1) provide a series of recommendations to the microbiological community to prevent the taxonomic misidentification of genome-sequenced fungal strains. We agree with the need for careful consideration in providing taxonomic information for an isolated strain and broadly support the authors’ recommendations. In their letter, the authors provide a table listing 141 strains within the order *Eurotiales* that they claim are misidentified and/or have not been deposited in a public culture collection. They included an organism for which we published the full draft genome sequence in *Microbiology Resource Announcements* (MRA) (2), claiming that this isolate was incorrectly identified and not deposited in a public culture collection. We do not completely disagree with their position; however, we felt compelled to add important nuance to the broader discussion raised by Houbraken et al. toward taxonomy and the release of open genomic data to the community at large.

One recommendation proposed by Houbraken et al. was to “perform an identification using the latest taxonomic insights. If needed, contact a taxonomist who can advise on the current identity of the strain.” While a laudable goal, not every research group has the luxury of ready access and funding to support a fungal taxonomist. If we had conferred with a fungal taxonomist when we conducted the phylogenetic analysis of our isolate, we might have been advised to use the recently preferred genus name *Paecilomyces* instead of the anamorph *Byssochlamys* when providing taxonomic information for our isolate (3). Our initial taxonomic inferences about strain AF001 were made between 2014 and 2016, prior to the introduction of a single-name nomenclature system, in which *Paecilomyces* was given priority over *Byssochlamys* (3). Our genome was not published until 2018, and we argue that instead of being outdated, our nomenclature was simply incorrect. This is an unfortunate error on our part, and we accept that *Paecilomyces* has priority in naming over the previously published *Byssochlamys*. Thus, our isolate is more aptly named *Paecilomyces* sp. strain AF001.

Initial efforts to identify the taxonomic relevance of our isolate, AF001, were focused on amplification and sequencing of the internal transcribed spacer (ITS) region. Despite the use of several sets of PCR primers (4), we were unsuccessful in amplifying the ITS region of our isolate (5). This is not uncommon, given that this is a highly variable region (6), which is also the reason that it can be a useful primary fungal barcode (7). Instead, we amplified portions of the small (18S) and large (28S) subunit rRNA genes and used the resulting sequence data in a phylogenetic analysis that placed our strain within a clade containing sequences for strains within the then-still-recognized genus *Byssochlamys* (8). The sequences from our isolate were 99% similar to those from an isolate named Byssochlamys spectabilis. Although rRNA gene sequence data are phylogenetically informative, the level of resolution afforded is not always sufficient to provide reliable taxonomic detail at the genus and species level. Therefore, we chose to only infer the generic identity of our isolate without a more comprehensive phenotypic characterization.

Another recommendation by Houbraken et al. was that any genome for which a sequence is published in *Microbiology Resource Announcements* (MRA) should be deposited in at least two independent, geographically distinct culture collections (i.e., in different countries). Again, we agree that this would be ideal and is the *de facto* standard when submitting a culture with the intent of complete characterization and naming in journals such as the *International Journal of Systematic and Evolutionary Microbiology* (IJSEM). However, the phenotypic analyses required to allow the deposition of strains in a public culture collection and their publication in the IJSEM were simply outside the scope of the studies we conducted. Furthermore, MRA specifically states in their information for authors that manuscripts providing an in-depth or comparative analysis of these resources will not be considered. We believe that the very existence of MRA is to provide an avenue for rapidly and openly disseminating microbiological resources that would otherwise not be published or made available. Our published genome added much-needed fungal genomic data for this part of the phylogenetic tree. The value of our published data is evidenced by its use by the authors to make their argument that our strain was incorrectly identified.

The claim that our isolate was incorrectly identified is the basis for the last point we would like to make. We took a very careful and conservative approach at the time to providing taxonomic information for our isolate. Our phylogenetic analyses based on rRNA gene sequences gave us confidence that our organism belonged among the other members of the genus *Paecilomyces* (we used the now-improper genus name *Byssochlamy*s). When the genome sequence data for our isolate were compared to the nearest neighbor, *Byssochlamys spectabili*s, it was only 84% similar based on average nucleotide identity (ANI). These were clearly different species, but with no other closely related genomes available for comparison, we decided to only attach the genus name, a requirement when submitting the genome to NCBI. The JGI Mycocosm project currently contains only 3 genomes from other species of *Paecilomyces*, the species Paecilomyces niveus and two strains of Paecilomyces variotii. Genome-level resolution among members of this genus and more broadly among the family *Trichocomaceae* is clearly needed, and even minimally characterized isolates such as those published within MRA will still help us resolve phylogenetic and taxonomic uncertainty.

Our conservative approach in taxonomically characterizing our isolate was taken because we would rather not incorrectly assign a species name to our isolate without sufficient data discerning it from other known and recognized taxa. Houbraken et al., on the other hand, have provided a “corrected identity” of our isolate as Paecilomyces dactylethromorphus in the supporting data for their letter. We are not fungal taxonomists and would otherwise defer to them in this matter; however, we feel that it is premature to assign a species-level taxonomic identity to an isolate based on the partial sequence of a single allele, the β-tubulin (BenA) gene, considering the lack of complete genome sequence data from other members of the genus. Fungal taxonomy in the genome era should arguably rely on comparisons of whole genomes (9). To do this, the community at large should be encouraged to publish genomic data of putative fungal strains rapidly and openly and not await a complete phenotypic characterization.

In summary, we applaud Houbraken et al. for providing some recommendations to address an ongoing problem with erroneous or incomplete taxonomic identification related to isolates that have had their genomes sequenced. Still, we think that a full phenotypic and genotypic analysis of these isolates prior to submission of these genome sequences runs counter to the stated purpose of MRA. Perhaps this should be reconsidered, but our current position is that this will slow or deter the rapid, open sharing of such resources. We also encourage the scientific community to show due diligence and caution in assigning taxonomic identities to their isolates. Further, our story points out that even what we considered a careful study of the literature can result in the perpetuation of dated or incorrect nomenclature. Despite these efforts and because of the rate of new data being generated regarding the immensely diverse microbial world, taxonomists will have their work cut out for them. Let us hope that they continue to strive to revise and organize these ever-growing collections of microorganisms.
